# In Response

**DOI:** 10.4269/ajtmh.14-0040b

**Published:** 2014-06-04

**Authors:** Max Grogl, Nestor Sosa, Mara Kreishman-Deitrick

**Affiliations:** Division of Experimental Therapeutics, Walter Reed Army Institute of Research, Silver Spring, Maryland. E-mail: max.grogl@gmail.com; Instituto Conmemorativo Gorgas de Estudios de la Salud, Avenida Justo Arosemena, Panama City, Panama; U.S. Army Medical Materiel Development Activity (USAMMDA), Fort Detrick, Maryland

Dear Sir:

Monge-Maillo and López-Vélez address the important issues of whether topical paromomycin treatments for cutaneous leishmaniasis (CL) achieve a higher cure rate when combined with another antileishmanial agent and the risk of mucosal leishmaniasis when cutaneous disease is treated locally.

We agree with these writers that combinations of paromomycin with other drugs are likely to be more effective than paromomycin alone. Although the writers focus on a combination with methylbenzethonium chloride (MBCL), we focus on a combination with gentamicin given significant adverse reactions noted with use of MBCL,^1^,^2^ strong animal data for paromomycin-plus-gentamicin,^3^ and the trend to increased efficacy from paromomycin-plus-gentamicin versus paromomycin-alone in the phase 2 study under discussion.^4^ When the data from our present phase 3 trial of paromomycin-plus-gentamicin (WR 279,396) versus paromomycin alone for *Leishmania panamensis* CL in Panama is known, others may wish to follow the suggestion of the letter writers and compare the most effective product in our trial to paromomycin-plus-MBCL but this undertaking should take into consideration the safety profile of these different formulations in addition to efficacy.

The issue of whether mucosal leishmaniasis consequent to CL is more likely after local therapy than systemic therapy is without a simple answer^5^ but we are following the World Health Organization (2010)^6^ current recommendation that local treatment is appropriate for all CL species, however, should be based on the benefit–risk considerations for each individual patient.

Finally, let us note that when we wrote that “Paromomycin 15% plus MBCL 12% has not been evaluated alone against *L. panamensis*,” we meant in a study in which only *L. panamensis* was documented to be the infecting parasite. For Krause and Kroger,^7^ only two parasites were identified as *L. panamensis*. For Armijos and others^8^ parasites were identified to the subgenus level but not to the species level.
